# The Combined Effect of Western Diet Consumption and Diclofenac Administration Alters the Gut Microbiota and Promotes Anastomotic Leakage in the Distal Colon

**DOI:** 10.3390/biomedicines12102170

**Published:** 2024-09-24

**Authors:** Melissa N. N. Arron, Stijn Bluiminck, Richard P. G. ten Broek, Thomas H. A. Ederveen, Lindsay Alpert, Olga Zaborina, John C. Alverdy, Harry van Goor

**Affiliations:** 1 Department of Surgery, Radboud Institute for Health Sciences, Radboud University Medical Center, 6525 GA Nijmegen, The Netherlands; 2 Department of Medical BioSciences, Radboud University Medical Center, 6525 GA Nijmegen, The Netherlands; 3 Department of Pathology, University of Chicago, Chicago, IL 60637, USA; 4 Department of Surgery, University of Chicago, Chicago, IL 60637, USA

**Keywords:** anastomotic leakage, Western diet, diclofenac

## Abstract

Background: Obesity, Western diet (WD) consumption, and the use of non-steroidal anti-inflammatory drugs (NSAIDs) are co-occurring and modifiable factors associated with microbiome dysbiosis and anastomotic leakage. We studied the combined effect of a Western-type diet (WD) and diclofenac, a standard NSAID used in surgical patients, on anastomotic healing and gut microbiota composition following distal colon resection. Methods: Forty-two rats were fed a WD for 6 weeks, after which they were randomized to either parenteral diclofenac 3 mg/kg/day or saline started on the day of surgery and continued for three days. The surgical procedure involved distal colon resection with anastomosis. Animals were sacrificed on postoperative day (POD)-3 or POD-5. Anastomotic healing was assessed and correlated with diclofenac treatment and gut microbiota composition, analyzed by 16S rRNA marker gene amplicon sequencing. Mucosal integrity of the anastomosis was evaluated by histological analysis. Results: Anastomotic leakage rate was 100 percent (8/8) in diclofenac-treated rats and 10 percent (1/10) in saline-treated controls on POD-5. Diclofenac administration in WD-fed animals induced a shift in microbiota composition, characterized by an increase in microbiota diversity on POD-5 and a significant 15-fold, 4-fold, and 16-fold increase of Proteobacteria, Bacteroidetes, and Verrucomicrobia, respectively. Diclofenac use in WD-fed animals caused mucosal erosion on POD-5, a phenomenon not observed in control animals. Conclusions: Consumption of a Western diet combined with diclofenac administration shifts the microbiota composition, associated with clinically relevant AL in the distal colon of rats.

## 1. Introduction

Anastomotic leakage (AL) occurs in 5–20% of all colorectal surgeries and is linked to severe morbidity and high mortality rates [1,2]. Despite extensive research, understanding the pathophysiology of AL has remained elusive. Although many clinicians attribute the mechanism of AL to be a result of a technical (suturing) failure by the surgeon, inadequate blood supply to the transected intestinal edges, or to increased tension on the anastomosis, research increasingly points towards causes independent of the surgeon’s experience and technique [3]. 

Research over the past decades has demonstrated that the pathophysiology of AL is multifactorial and impacted by perioperative care and patient characteristics. More recently, a role has emerged for the gut microbiome in the development of AL [4,5,6]. Although our understanding of the underlying mechanisms and involved pathogens is still in the early stages, maintenance of the delicate balance between the health-promoting microbiota and overgrowth of resistant and virulent microbial pathogens is believed to be essential for the uncomplicated healing of anastomoses. Disruptions in this balance can occur due to various patient-related and perioperative factors, contributing to impaired anastomotic healing [7,8]. The patient’s diet and perioperative analgesia appear to be modifiable factors known to affect gut microbiome, and both are associated with AL. 

Diclofenac, a non-steroidal anti-inflammatory drug (NSAID), is frequently administered to patients in enhanced recovery programs after surgery (ERAS) to provide postoperative analgesia as an opioid-sparing measure. However, NSAID administration after colorectal cancer surgery has been debated for years due to its potential disruptive effect on anastomotic healing. Previous experimental studies have demonstrated that diclofenac can have a detrimental effect on anastomotic healing; however, the pathophysiology remains to be elucidated [9,10]. Additionally, a recent study from our research group demonstrated that the administration of diclofenac results in microbiota changes associated with the development of AL (our preliminary study).

In addition to diclofenac administration, consumption of a high-fat obesogenic Western diet (WD) and the consequences of obesity are modifiable factors that can alter gut microbial composition and increase the risk of developing AL [11,12]. Of particular concern is the effect of a WD on the proliferation and expression of a collagenolytic phenotype in *Enterococci*, a normal commensal gut inhabitant that can shift from a probiotic to a pathogenic state depending on the environmental context [13]. Notably, diclofenac is known for its antibacterial effects on *Enterococcus faecalis* in vitro [14]. Given this, it is crucial to consider the interplay between diclofenac and dietary habits, hypothesizing that administration of an immune modulatory drug like diclofenac, when combined with consumption of a WD, may have unanticipated effects on the gut microbiome, leading to microbiome-mediated AL.

This study aims to investigate the combined effect of obesity, consumption of a western diet, and NSAIDs on the gut microbiota and the development of anastomotic leakage with the goal of advancing an understanding of the association between microbiome alterations and the development of AL. Given that both the use of diclofenac and the consumption of a WD are potentially modifiable factors in surgical patients, understanding their additive or synergistic effect on AL is relevant for current clinical practice. 

## 2. Materials and Methods

### 2.1. Animals

Male Wistar rats aged 8–10 weeks (Charles River Laboratories, Raleigh, NC, USA), with an average weight of 230 g (range 160 to 305 g), were used and maintained according to Institutional Animal Care and Use Committee #72589. Rats were acclimated to laboratory conditions for one week prior to experimentation. Afterwards, the standard high-fiber low-fat diet (Envigo 2018 Global Diet Rodents) was replaced by an obesogenic Western diet (WD, 60 kcal% fat, BioServe S3282) and their weight was monitored weekly (see Supplementary Table S1). Rats were allowed free access to food and water. They were housed by twos under standard laboratory conditions in a temperature-controlled room (22–24 °C) with a 12 h light–dark cycle. The cages were enriched with nesting material and were randomly placed in the rack. All experiments were performed in accordance with the National Institutes of Health guidelines, and approval was obtained from the University of Chicago Animal Care and Use Committee (Protocol 71744).

### 2.2. Groups

**Figure 1:**
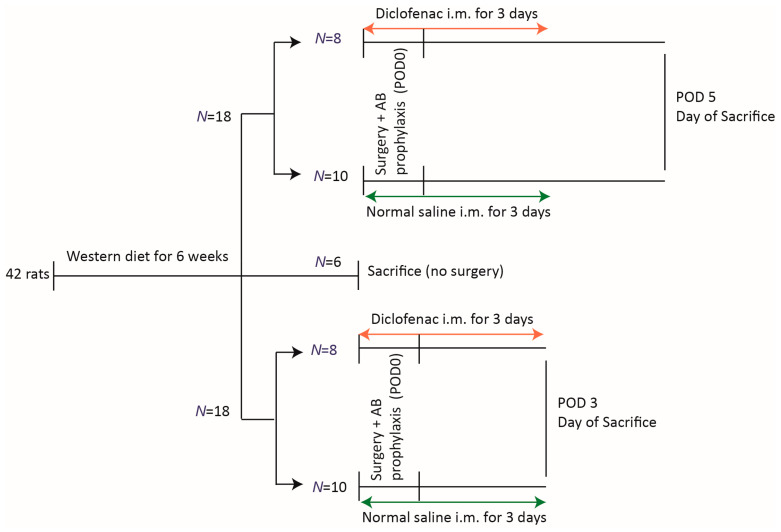
Experimental design. The orange arrow (diclofenac) and the green arrow (normal saline) indicate the treatments provided. POD, postoperative day; i.m., intramuscular.

All animals in this study received 6 weeks of WD. Afterward, six rats were sacrificed, and samples of fecal content and anastomotic tissue were obtained to determine their baseline preoperative microbiota status. The remaining 36 animals were randomized in two groups to receive either diclofenac (*N* = 16) (Spectrum Chemical, New Brunswick, NJ, USA) or saline (control, *N* = 20) and subsequently underwent surgery (Figure 1). Randomization was done per cage, using a computer-assisted random number generator. To study anastomotic healing and gut microbiota composition over time, animals were sacrificed at two time points: postoperative day (POD)-3 and POD-5. 

### 2.3. Surgical Technique and Diclofenac Administration

Thirty minutes before laparotomy, rats received a subcutaneous injection of cefoxitin (40 mg/kg, West-Ward Pharmaceutical Corp, Eatontown, NJ, USA), a common second-generation cephalosporin used in colorectal surgery. The rats were anesthetized by inhalation of 3% isoflurane (Henry Schein, Dublin, OH, USA, mixed with pressurized air and oxygen. Their skin was shaved and disinfected with 70% ethanol. Body temperature was kept at 38 °C by a heating lamp and surgical drapes. A 3 cm midline abdominal incision was made under sterile conditions and the distal colon was visualized. The intestine was protected from desiccation by gauzes soaked in 0.9% NaCl. The colon was transected and at 3 cm proximal to the peritoneal reflection a 1 cm resection was performed, and continuity of the bowel was restored with an inverted single layer anastomosis made by eight interrupted 8–0 Ethilon (Ethicon) sutures. Anastomotic integrity was confirmed by injecting saline into the rectum and visually inspecting the anastomosis for leaks. The abdominal wall was closed in two layers with a running suture (Vicryl 3–0, Ethicon). Rats were administered a bolus of 10 mL 0.9% NaCl subcutaneously for rehydration. Diclofenac sodium (Spectrum Chemical, New Brunswick, NJ, USA) was dissolved in 0.1% polysorbate in saline and was given in a dose of 3 mg/kg/day, in two equal doses intramuscular for three days, starting on the day of surgery. Rats were maintained on the WD until sacrifice. Animals were checked twice daily after surgery for signs of distress and pain (hunched back posture, ruffled fur, or no response to touch). Humane endpoints were defined for early removal of animals from the experiment to avoid unnecessary suffering.

At POD-3 and POD-5, animals were euthanized using CO_2_ asphyxiation, followed by relaparotomy to evaluate anastomotic healing and to collect fecal content and colonic anastomotic tissue for subsequent microbiota analyses and histology.

### 2.4. Outcome Parameters 

#### 2.4.1. Anastomotic Healing

Anastomotic healing was macroscopically scored using a validated anastomotic healing score (AHS): AHS 0, normal healing; AHS 1, flimsy adhesions; AHS 2, dense adhesions without abscess; AHS 3, small abscess either with or without adhesions; AHS 4, large abscess either with or without adhesions; AHS 5, visible dehiscence or gross leak with peritonitis [15,16]. The AHS 4 and AHS 5 are considered a clinically relevant AL. The scoring was done independently by two researchers (M.N.N.A. and S.B.) and any differences in scores were solved by consensus. 

#### 2.4.2. Microbiota Analysis: Sample Collection

For longitudinal analysis of the microbiota, expelled fecal pellets were collected at baseline (one week after accommodation at the facility), six weeks after the start of the WD, and at sacrifice (POD-3 and 5). In addition, at sacrifice, fecal content (snap frozen) from the anastomotic site and a 0.5 cm section of anastomotic tissue were collected for microbial DNA isolation to measure fecal and tissue-adhered luminal microbes.

#### 2.4.3. Microbiota Analysis: DNA Isolation

Whole fecal pellets (±1 g) and 0.25 g of anastomotic tissue were homogenized with the FastPrep-24 5G Instrument (MP Biomedicals, Santa Ana, CA, USA). Total microbial genomic DNA was extracted from the homogenate with the PowerFecal DNA Kit (Qiagen, Hilden, Germany), according to the manufacturer’s instructions. 

#### 2.4.4. Microbiota Analysis: 16S Ribosomal RNA Marker Gene Library Preparation and Sequencing 

Total microbial genomic DNA obtained from the rat samples was used as template DNA. Each 25 μL PCR reaction contained 10 μL Platinum Hot Start PCR Master Mix (ThermoFisher, Waltham, MA, USA), 13 μL of PCR-grade water (Sigma-aldrich, Saint Louis, MO, USA), 0.5 μL forward primer (10 μM), 0.5 μL Golay Barcode Tagged Reverse Primer (10 μM), and 1 μL template DNA. The PCR conditions were 94 °C for 3 min followed by 35 cycles at 94 °C for 45 s, 50 °C for 60 s, and 72 °C for 90 s, with a final extension of 10 min at 72 °C. The amplicons were cleaned, pooled, and quantified using the Quant-iT PicoGreen (Invitrogen, Carlsbad, CA, USA) double-stranded DNA assay kit following EMP (Earth Microbiome Project) benchmarked protocols. Pooled amplicons were then pair-end sequenced (2 × 150 base pairs) on an Illumina (San Diego, CA, USA) MiSeq sequencing run at the Argonne National Laboratory [17]. Then, 16S rRNA marker gene Illumina sequencing of the bacterial V4 region using 515F (5′ GTGYCAGCMGCCGCGGTAA 3′) and 806R (5′ GTGCCAGCMGCCGCGGTAA 3′) primer appended with Illumina adaptor sequences was performed. The FASTQ paired-end reads, as received from the sequence facility, were assembled with PEAR into FASTA pseudoreads by using strict assembly settings: quality threshold 30, minimum overlap 35, and *p*-value 0.0001 [18].

For generation of the microbiota-to-sample compositional matrix, a customized Python workflow based on Quantitative Insights Into Microbial Ecology (QIIME, v1.8) was adopted (https://qiime2.org/, accessed on 1 January 2020) [19]. Reads were filtered for chimeric sequences using the UCHIME algorithm, as implemented in QIIME [20]. The operational taxonomic units (OTU) clustering (open reference OTU picking), taxonomic assignment, and reference alignment was done with the ‘pick_open_reference_otus.py’ workflow script of QIIME, using UCLUST (USEARCH) [21] as the clustering method (97% identity) and Greengenes version 13.8 as a 16S reference database [22]. OTUs consisting of only a single sequence were removed. Sequences that could not be aligned by PyNAST [23] against the 16S reference alignment were removed. Hierarchical clustering of samples was performed using an unweighted pair group method with arithmetic mean (UPGMA) with weighted UniFrac as a distance measure, as implemented in QIIME. Alpha diversity metrics (PD whole tree, Chao1, Observed Species, and Shannon) were calculated by bootstrapping and taking the average over 10 trials. Bacterial composition analysis was conducted on the genus level. NOTE: Due to technical limitations in the resolution of 16S marker gene sequencing, OTU classification on the level of species should be interpreted with caution. 

#### 2.4.5. Histology and Staining 

The effect of diclofenac on anastomotic healing of the colonic mucosa was further investigated by histologic evaluation. The distal colon including the anastomosis was resected at 0.5 cm above and below the suture line, fixed in 10% formalin overnight, and washed afterwards with 70% ethanol. Samples were embedded in paraffin with the suture line centered, cut in 5 µm slices, and stained with hematoxylin and eosin (H&E). An independent pathologist evaluated the histological slides in a blinded manner and scored them according to standardized histopathological examination: neutrophilic inflammation (0 = none, 1 = mild, and 2 = severe), eosinophilic infiltrate (0 = none, 1 = mild, and 2 = moderate/severe), basophils (0 = not identified, 1 = rare, and 2 = numerous), ulceration (0 = none, 1 = small, and 2 = large), and granulation tissue (1 = mild to moderate, and 2 = abundant).

### 2.5. Data Analysis 

The following comparisons were performed for microbiota composition and histology, regarding the outcomes of AL: (1) diclofenac-treated versus control animals on POD-3; (2) diclofenac-treated versus control animals on POD-5; (3) diclofenac-treated animals on POD-3 versus diclofenac-treated animals on POD-5; and (4) control animals on POD-3 versus control animals on POD-5. 

To study microbiota alterations over time, expelled fecal microbiota-derived data was analyzed at four moments: baseline, 6 weeks after start of WD (preoperative), and at POD-3 and 5. Subsequently, at POD-3 and 5, the microbiota data of the anastomotic tissue were analyzed.

### 2.6. Statistical Analysis

The required sample size was determined with expected leak rates of 75% in the diclofenac group and 0–10% in the control group, based on previous studies [10]. Using an alpha of 0.05 and a beta of 0.8, this resulted in 8–10 animals per study group.

Statistical analysis of the AHS was performed using one-way ANOVA with Bonferroni (BF) correction for multiple comparisons. For univariate analysis of microbial taxa, the non-parametric Mann–Whitney U test (MWU) was used to compare groups. For multivariate analyses, redundancy analysis using Canoco v.5.04 was performed [24]. Redundancy analysis (RDA) calculates *p*-values by randomly permuting the sample status and by thereafter counting the number of times that a permuted set of samples had a better separation than the original one. Redundancy Analysis Plots were made to visualize microbiota compositional differences between groups. Results are presented as means with corresponding standard deviations. A *p*-value less than 0.05 was considered statistically significant. Statistical analysis was performed using GraphPad Prism 8 and SPSS Statistics v.25.

## 3. Results

### 3.1. Animal Welfare

**Figure 2:**
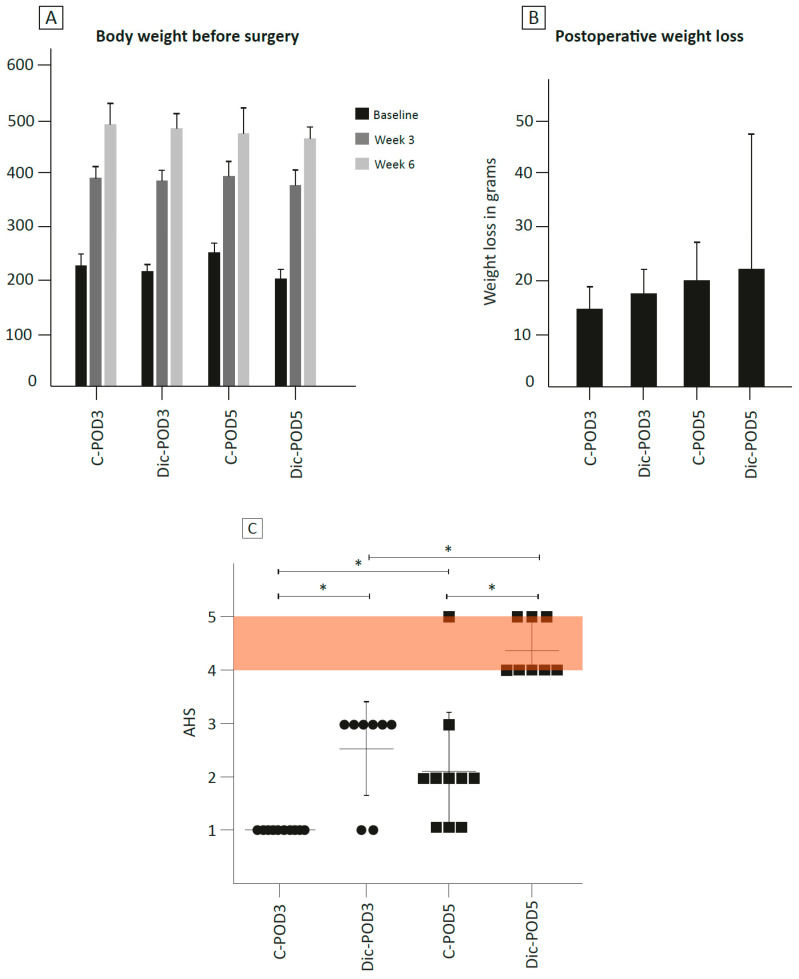
Effect of WD on body weight and anastomotic healing score and weight loss at sacrifice. (**A**) Mean (s.d.) body weight in grams of rats during 6 weeks on Western diet. C, control; Dic, diclofenac. (**B**) Mean (s.d.) postoperative weight loss at sacrifice. (**C**) Anastomotic healing score (AHS, mean with s.d.) on postoperative day 3 and postoperative day 5. Shaded orange area indicates a clinically relevant leak (AHS 4, large abscess or AHS 5, dehiscence or peritonitis). Individual data points at POD-3 are indicated with circles, while data points at POD-5 are indicated with squares. * indicates *p* < 0.05 (*T* test). C, control; POD, postoperative day; Dic, diclofenac.

All 42 animals were administered a Western diet (WD) for six weeks, during which they achieved, on average, a weight of 260 ± 33 g, effectively doubling their weight compared to baseline (Figure 2A). Following surgery, 16 animals were randomized to receive diclofenac, while 20 received normal saline for 3 days postoperatively. During the 5-day postoperative period, animals lost an average of 20 ± 12 g (4%) in weight, without significant differences between groups (Figure 2B). None of the animals died prematurely or were removed due to reaching the humane endpoint.

### 3.2. Anastomotic Healing 

Animals fed a WD and subjected to diclofenac exhibited significantly higher (i.e., worse) anastomotic healing scores (AHS) and signs of clinically relevant leakages (defined as AHS 4 or 5). On POD-3, WD-fed animals subjected to diclofenac had significantly (*p* < 0.01) higher AHS (2.5 ± 0.9) compared to WD-fed controls (1.0 ± 0, Figure 2C). No animals in this group had a clinically relevant AL on POD-3. On POD-5, all (8/8) WD-fed animals subjected to diclofenac showed macroscopic signs of clinically relevant leakage, compared to one (1/10) WD-fed control animal. When examining WD-fed controls, no animals on POD-3 and only one (1/10) animal on POD-5 developed clinically relevant AL. However, on POD-5, the AHS was significantly higher compared to POD-3 (*p* < 0.05). 

### 3.3. The Effect of Diclofenac on Colonic Mucosa

**Figure 3:**
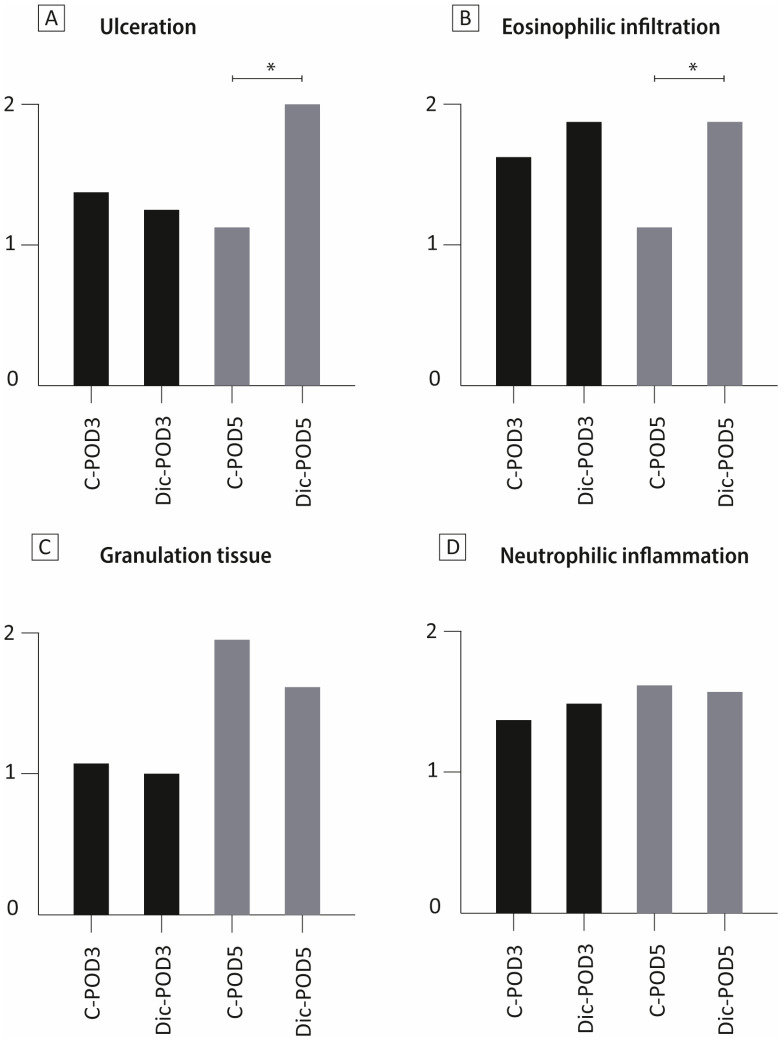
Histological examination of the mucosal integrity of the anastomosis. Four items were assessed at histological examination: (**A**) ulceration (0 = none, 1 = small, and 2 = large), (**B**) eosinophilic infiltration (0 = none, 1 = mild, and 2 = moderate/severe), (**C**) granulation tissue (1 = mild to moderate, and 2 = abundant), and (**D**) neutrophilic inflammation (0 = none, 1 = mild, and 2 = severe). C, control; POD, postoperative day; Dic, diclofenac.

Diclofenac administration affected the epithelial structure through ulceration and increased eosinophilic infiltration in animals sacrificed on POD-5, as observed by H&E staining (Figure 3A,B). These changes were not observed in animals sacrificed on POD-3. Overall, histologic scores on the presence of granulation tissue and neutrophilic inflammation revealed no differences between groups (Figure 3C,D). 

### 3.4. The Effect of WD and Surgery on Microbial Diversity and Composition (No Diclofenac)

**Figure 4:**
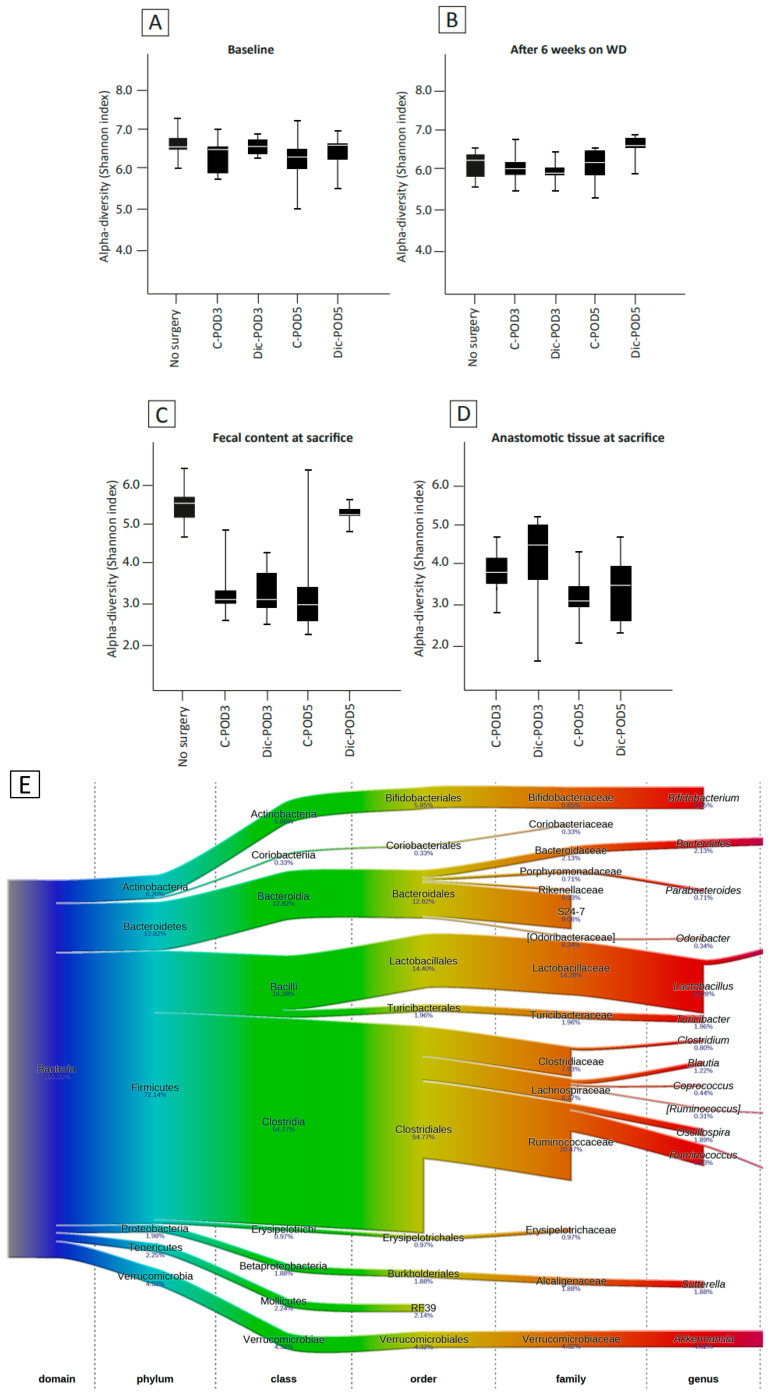

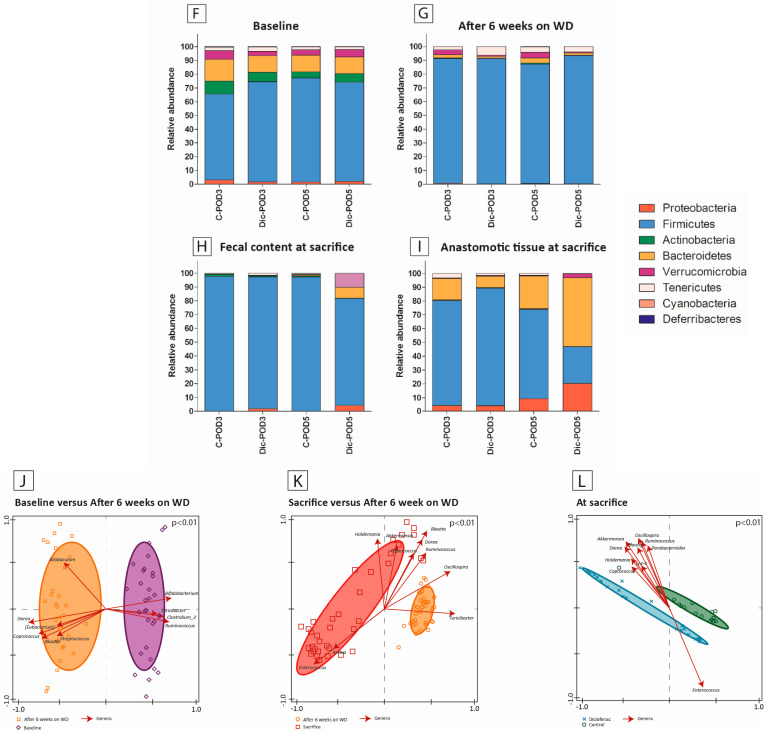
16S ribosomal RNA analysis of microbiota in fecal content and anastomotic tissue. (**A**–**D**): Comparison of alpha-diversity (Shannon index) between groups at baseline (**A**), after six weeks on Western diet (**B**), fecal content at sacrifice (**C**), and anastomotic tissue at sacrifice (**D**). Mean value (white line) with interquartile range (box). (**E**) Overall microbiota composition (on average, all samples). (**F**–**I**): Relative abundance plots at phylum level at baseline (**F**), after 6 weeks on Western diet (**G**), fecal content at sacrifice (**H**), and anastomotic tissue at sacrifice (**I**). C, control; POD, postoperative day; Dic, diclofenac. (**J**–**L**): Redundancy analysis (RDA) of fecal content at baseline (purple) and after six weeks on Western diet (orange) (**J**), at sacrifice (red), and at 6 weeks on Western diet (orange) (**K**), and diclofenac-treated (blue) and control (green) at sacrifice (**L**). Red arrows represent the 10 best-fitting genera (names in Italics), which are genera best explaining the compositional differences.

After six weeks of consuming a WD, there was a loss of microbial diversity in the fecal content (*p* = 0.02, Figure 4A,B). WD-fed animals had a significant shift at the phylum level, characterized by a bloom of Firmicutes (70.9% to 90.5%, *p* < 0.001) and a decrease of Proteobacteria (2% to 0.4%, *p* < 0.001), Actinobacteria (6.7% to 0.4%, *p* < 0.001), and Bacteroidetes (13.0% to 2.2%, *p* < 0.001) (Figure 4G). Redundancy analysis (RDA) revealed significant separation of the gut microbiota before and after six weeks of WD (*p* < 0.01, Figure 4J), characterized by a significant increase in *Blautia* (1.2% to 5.3%, *p* < 0.001) and *Allobaculum* (0.01% to 3.8%, *p* < 0.001) and a significant decrease in *Bifidobacterium* (6.3% to 1.7%, *p* < 0.001), *Ruminococcus* (5.7% to 3.6%, *p* < 0.001), and *Akkermansia* (4.6% to 2.4% *p* < 0.001). The most abundant genus after 6 weeks of WD was *Lactobacillus* at 16.3% (compared to 14.2% at baseline, *p* = 0.18).

Postoperative assessment of the microbiota revealed a significant loss of microbial diversity in the fecal contents of all groups at the time of sacrifice compared to the preoperative levels (*p* < 0.01, Figure 4B,C). Subsequent analysis showed that the phyla Firmicutes (91.9%), Bacteroidetes (2.1%), Verrucomicrobia (2.7%), and Proteobacteria (1.6%) were the most abundant phyla at sacrifice. RDA revealed a significant separation of the gut microbiota at the time of sacrifice compared to preoperatively (*p* < 0.01, Figure 4K). This was characterized by a bloom of *Enterococcus* (0.5% to 26.3, *p* < 0.001) and *Eubacterium* (1.1% to 4.4%, *p* = 0.002).

### 3.5. The Effect of WD, Surgery and Diclofenac on Microbial Diversity and Composition

Diclofenac administration in WD-fed animals was associated with significantly increased microbial diversity on POD-5 (*p* < 0.01, Figure 4C), but not on POD-3. On POD-5, there was a bloom of Proteobacteria, Bacteroidetes, and Verrucomicrobia compared to the control, with 15-fold, 4-fold, and 16-fold increases respectively (*p* < 0.01, *p* = 0.02, *p* = 0.01, Figure 4H). RDA revealed significant separation of microbiota composition among animals that received diclofenac versus controls on POD-5 (*p* < 0.01, Figure 4L). This was characterized by significant higher relative abundance of *Blautia* (10.6% vs. 0.8%), *Akkermansia* (10.2% vs. 0.4%, *p* = 0.007) and a lower relative abundance of *Enterococcus* (29.6% vs. 2.1%, *p* < 0.001), and *Allobaculum* (5.5% vs. 1.4%, *p* = 0.470). On POD-3, we observed a significantly lower abundance of *Lactobacillus* among diclofenac-treated animals (10.6% vs. 25.05%, *p* = 0.027); however, this difference was not significant on POD-5 (5.1% vs. 10.1%, *p* = 0.326).

Among animals treated with diclofenac, we noted ongoing microbiota changes from POD-3 to POD-5, despite diclofenac being administered only for the first 3 days postoperatively. There was an increase in microbial diversity from POD-3 to POD-5 (3.4 ± 0.6 versus 5.2 ± 0.2, *p* < 0.01, Figure 4C). Further analysis revealed a reduction in Firmicutes (95.5% to 77.3%, *p* < 0.001) and an increase in Bacteroidetes (0.08% to 7.6%, *p* = 0.023) and Proteobacteria (1.9% to 4.3%, *p* = 0.29). At the genus level, a significant decrease in *Enterococcus* (34.1 to 2.1%, *p* = 0.003) was observed, alongside a notable bloom of *Akkermansia* (0.08% to 10.2%, *p* = 0.010), *Blautia* (0.64 to 10.6%, *p* < 0.001), and *Dorea* (1.0 to 5.6%, *p* < 0.001) at POD-5 compared to POD-3.

### 3.6. Microbiota Composition Differences between Anastomotic Tissues and Fecal Contents

The microbial diversity of the fecal luminal contents was similar to the microbiota in the anastomotic tissue in either control rats or diclofenac rats sacrificed on POD-3. There was a significant difference in microbial diversity of diclofenac rats on POD-5, with less microbial diversity at the anastomotic tissue, compared to fecal content and compared to controls (5.2 ± 0.2 versus 3.4 ± 0.9, *p* < 0.01, Figure 4C,D). Compared to fecal content, tissue overall had higher relative abundance of Bacteroidetes (24.3% vs. 2.1% (*p* < 0.001) and Proteobacteria (9.4% vs. 1.6%, *p* < 0.001), and a lower abundance of Firmicutes (63.3% vs. 91.9%, *p* < 0.001) (Figure 4I). When comparing microbiota at the anastomosis of diclofenac-treated animals and controls, we observed no difference in microbial diversity at the anastomotic tissue at POD-3 and 5. However, we observed compositional changes on RDA characterized by an increase in *Bacteroides* (46.9% vs. 21.9%, *p* = 0.077), *Enterococcus* (3.2% vs. 16.8% in controls, *p* = 0.016), and *Akkermansia* (2.8% vs. 0.4% in controls, *p* = 0.278) among diclofenac-treated animals. 

### 3.7. The Effect of Diclofenac on the Presence of Enterococcus

**Figure 5:**
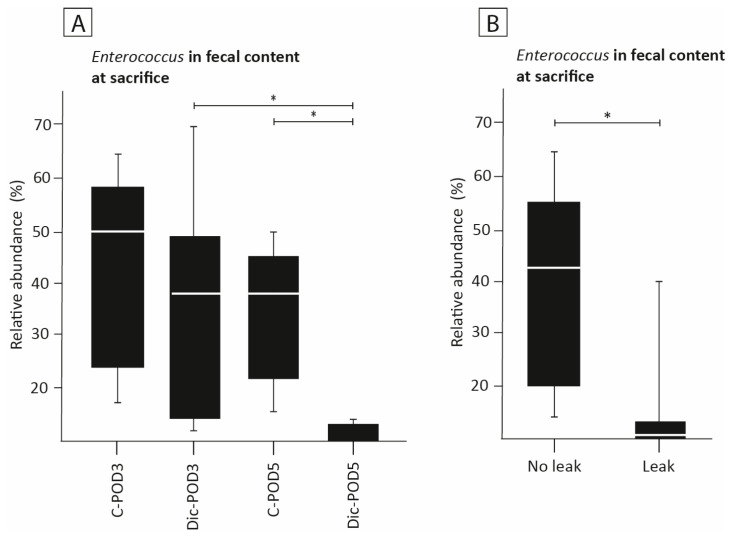
16S ribosomal RNA analysis of *Enterococcus*. (**A**,**B**): Comparison of relative abundance of *Enterococcus* in fecal samples at sacrifice (**A**) between study groups and (**B**) between animals that had a clinically relevant leak (AHS 4 or 5) and animals that did not have a clinically relevant leak (AHS ≤ 3). Mean value (white line) with interquartile range (box) and range (error bar). * indicates a *p* < 0.05. C, control; POD, postoperative day; Dic, diclofenac.

The relative abundance of *Enterococcus* in the fecal content of animals on POD-3 was not significantly different between diclofenac and control animals (34% vs. 44%, Figure 5A). In animals sacrificed on POD-5, diclofenac administration was associated with a significantly reduced abundance of *Enterococcus* in fecal contents compared to controls (1.9% vs. 34%, *p* < 0.01, Figure 5A). At POD-3, this difference was not significant (34% vs. 44%). The abundance of *Enterococcus* decreased significantly from POD-3 to POD-5 among diclofenac-treated animals (32.5% vs. 3.1%, *p* = 0.006).

In anastomotic tissue, the abundance of *Enterococcus* was also significantly lower among diclofenac-treated animals (3.2% vs. 16.8% *p* = 0.016). When assessing the abundance of *Enterococcus* in relation to development of AL, we observed that developing AL was associated with a reduced abundance of *Enterococcus* in the luminal fecal contents (7% vs. 38%, *p* < 0.01, Figure 5B). Developing AL was also associated with a reduced abundance of *Enterococcus* (3.1% vs. 32.5%) in the anastomotic tissue, compared to control (*p* = 0.016). This association was not shown at POD 3 (32.5% vs. 26.4, *p* = 0.517). 

## 4. Discussion

Here we demonstrate that postoperative administration of diclofenac in rats consuming a Western diet (WD) impairs distal colonic anastomotic healing, resulting in clinically relevant anastomotic leakage. Furthermore, we showed the effect of diclofenac on the gut microbiota in animals subjected to both a WD and colonic surgery. Previous studies have shown AL after administration of diclofenac; however, this is the first to explore the combined effect of diet and the immune-modulatory drug, diclofenac, on the gut microbiota in relation to AL [9,10,16]. These results are important as they suggest that this combination may lead to a diet-induced, microbiota-mediated development of AL. Given that diet has been shown to have the most profound effect on the gut microbiome, the extent to which a patient’s dietary history influences anastomotic healing, especially in the current era of the use of immunomodulatory agents such as diclofenac and biologics, is significant. 

The finding in this study is in contrast with prior studies of Yauw et al. in which diclofenac administration in standard diet-fed animals was associated with AL in the proximal but not the distal colon [10]. In the current study, we observed leakage in the distal colon among animals treated with diclofenac, regardless of whether they were fed a Western diet or a standard diet (data of standard diet-fed animals are not shown). There are several explanations for this: Firstly, in the study by Yauw et al., anastomotic healing was only assessed on POD-3, while the current study, anastomotic healing developed at POD-5 [10]. Secondly, in the current study, all animals received antibiotic prophylaxis with cefoxitin to mimic its use in clinical practice. Previous studies showed that antibiotic administration in combination with NSAIDs can cause severe peritonitis and small intestinal perforation in mice [25]. It is hypothesized that this is a bacteria-mediated effect, since germ-free mice do not develop these mucosal defects when NSAIDs only are administered [26]. Thirdly, this could be an example of the synergistic effects of WD and the use of diclofenac, whereby there is both induction of mucosal damage from the drug and alterations in the microbiome—a “two-hit hypothesis of infection” in which a severe primary insult, in the presence of an altered microbiome, advances to further damage and disruption in anastomotic healing [27]. In the current study, there is a ‘’triple-hit’’: animals are fed an WD, receive antibiotic prophylaxis, and are treated with diclofenac. All treatment exposures are known to have immunomodulatory and microbiome-mediated effects; therefore, it may not be unexpected to observe an impairment in anastomotic healing. 

Administration of WD was associated with significant changes in the gut microbiota. After six weeks of consuming WD, the gut microbiota showed a reduced microbial diversity and the elimination of *Bifidobacterium*. Low microbial diversity is associated with poor health conditions [28,29]. Furthermore, the genus *Bifidobacterium* is considered to have health-promoting effects [30,31]. This gut microbiota dysbiosis may therefore have contributed to a disturbed microbial environment, resulting in poor conditions for anastomotic healing. 

Not only did WD consumption shift the composition of the gut microbiota, but diclofenac administration was also associated with a significant disruption of the gut microbiome, characterized by an increased relative abundance of Proteobacteria and Bacteroidetes and a decreased relative abundance of Firmicutes in fecal contents. These results are in line with previous studies investigating the effect of diclofenac on microbiota composition in rats [32,33].

Administration of diclofenac in WD-fed animals was associated with a significant increase in microbial diversity. This finding is in contrast with previous studies showing marked microbial changes from NSAIDs, but no or little impact on overall diversity [34]. Increased diversity is generally believed to contribute to greater stability of an individual’s microbiome and prevent pathogenic bacteria, which contribute to AL, from blooming [35,36,37,38]. However, in previous studies, the effect of diclofenac on the microbiome was studied in the absence of the process of surgery (anaesthetic agent, tissue trauma, and anastomotic surgery) and the consumption of a WD. A potential explanation for the increased microbial diversity observed in our model following diclofenac administration is that diclofenac induces a redistribution of microorganisms within a microbiome already significantly affected by the WD and surgery. Further work is underway to confirm this assertion.

Results from this study have generated several hypotheses to be tested to determine the mechanisms by which AL develops in WD-fed animals subjected to diclofenac. First, a direct topical impact of toxic metabolites from diclofenac could occur. In the current study, AL does not develop until POD-5. This might be due to delayed presentation of diclofenac metabolites at the anastomosis. Previous studies have shown that more proximal parts of the intestinal tract, such as the terminal ileum and proximal colon, are exposed to the highest concentrations of diclofenac metabolites [39]. This is the result of re-absorption of bile components such as diclofenac metabolites (diclofenac-acyl-glucuronide) in the terminal ileum. Before reaching the distal colon, part of the diclofenac metabolites are already reabsorbed. However, reactivation of diclofenac-acyl-glucuronide in the gut, through cleavage by bacterial glucuronidase, causes the accumulation of harmful molecules that can still reach the distal colon and threaten colonic integrity, albeit at a later time. 

Secondly, administration of diclofenac in WD-fed animals was associated with significantly increased colonic mucosal ulceration on POD-5. This suggests that NSAID-induced ulceration is involved in AL and that its pathophysiology might correspond with the ulcerative effect of NSAIDs on the gastric mucosa, initially causing superficial erosion and evolving to ulcers with time and repetitive doses of NSAIDs [40]. Although NSAIDs are mainly known for their effect on the gastric mucosa, there is increasing evidence for mucosal injury of the distal gastrointestinal tract [41,42]. These mucosal lesions seem to be the result of COX-1 inhibition, which is known to be responsible for the cytoprotective effects on gastric mucosa and function. Interestingly, previous studies have posited that the detrimental effect of NSAID on anastomotic healing is based on COX-2 inhibition [10,43,44]. COX-2 is usually involved in pathological conditions such as inflammation; it has been hypothesized that COX-2 inhibition by NSAIDs inhibits inflammation and proliferation, which are essential in the early phase of wound healing [45]. Since diclofenac, a non-selective NSAID, inhibits both COX-1 and COX-2, it is possible that both above-described mechanisms contribute to disrupted anastomotic healing. 

Lastly, there might be a bacteria-mediated effect, as both the consumption of a WD and the administration of diclofenac exert distinct influences on the gut microbiota. Without the benefit of metabolomic data and more in-depth phenotyping of individual strains, microbiome data are limited. Therefore, the extent to which a given strain behaves as a commensal organism or a pathogenic one cannot be determined. However, supporting a bacteria-mediated effect is the observation that diclofenac administration in WD-fed rats was associated with increased ulceration at the anastomosis. In a recent study, we showed that the administration of diclofenac in rats fed a high-fiber, low-fat diet does not result in mucosal ulcerations (our preliminary study). This might suggest that diclofenac-induced ulceration is diet dependent. A potential explanation is that NSAID-associated colonic mucosal ulceration is dependent on the local microbiome environment. This is supported by studies showing that normal commensal bacteria can exert pathological features if the intestinal environment changes [15].

Another hypothesis for microbiome-mediated mucosal damage could be the depletion of the probiotic *Lactobacillus* in diclofenac-treated animals, as in the current study, *Lactobacillus* depletion was more pronounced in animals administered diclofenac. A role for the microbiome in NSAID-induced mucosal damage is supported by the finding that in a healthy microbiome, *Lactobacillus* exerts beneficial health effects, such as protection of the integrity of the colonic epithelium and cell junctions [46]. Experimental rat models have shown that administration of probiotics such as *Lactobacillus* and *Bifidobacterium* prevents NSAID-induced enteropathy [47,48,49]. 

Given that previous studies have posed that *Enterococcus faecalis* contributes to the pathophysiology of AL in mice, we critically assessed the *Enterococcus* genus [13]. While the high abundance of specific *Enterococcus Faecalis* strains was associated with AL in the previous study, in the current study, *Enterococcus* depleted in diclofenac-treated animals, which was associated with clinically relevant AL. The anti-bacterial effect of diclofenac upon *Enterococcus* could have contributed to this finding [14]. As previously mentioned, *E. faecalis*, depending on the strain and the in vivo phenotype expressed, can behave either as a probiotic commensal bacterium or a virulent pathogen. Interestingly, the fact that *Enterococcus* concentrations are high in diclofenac-treated animals on POD-3 and low in diclofenac-treated animals on POD-5 supports the previously posited mechanism of action of diclofenac: that there is potentially a delayed harmful effect of diclofenac on the colonic mucosa and microbiota. Nevertheless, our findings suggest that the proposed role of *Enterococcus* in the pathophysiology of AL needs will require further investigation. 

In our study, a WD alone did not result in clinically relevant AL, as only two of ten rats developed small abscess formation or gross leakage, which was similar to rats fed a normal high-fiber/chow diet (our preliminary study). This finding is in contrast with previous studies in mice showing clinically relevant AL after a WD [10,50]. A potential explanation is the interspecies difference, as rats are potentially more resilient and require more stress to develop AL than mice.

In addition to taxonomic analyses of the microbiome, this study explored the influence of diclofenac administration on microbiome function in WD-fed animals. Although no direct functional data were obtained, functional prediction analyses were conducted using the 16S rRNA OTUs with the PICRUSt2 software (V2.2.0.). This analysis identified 13 pathways that were significantly different between WD-fed animals that received diclofenac and those that did not. However, due to the low abundance and small effect sizes observed, these differences were not considered clinically relevant and are therefore not further discussed in this paper. Detailed analyses, including differential abundance analyses of KEGG pathways and KEGG Orthology, as well as PCA ordination analysis, are provided in Supplement File S2 [51,52].

There are some limitations to our study. QIIME was used rather than the more recent QIIMEII software (V1.8.) for 16S RNA throughput sequencing, and an operational taxonomic unit (OTU) based analysis was conducted instead of using amplicon sequence variants (ASVs) for the assessment of bacterial diversity. While it is considered that ASVs and QIIMEII provide more robust results, this is particularly true when assessing taxa below the genus level, which was not pursued in the current study. Moreover, 16S short-read Illumina data are very comparable between ASV and OTU, mainly because analyses were performed on the genus level. Therefore, the results presented are believed to be accurate and biologically relevant. Our data were normalized by calculating relative abundances (by total-sum scaling). We acknowledge that performing differential abundance analysis statistics on compositional data (i.e., relative abundance data) has its limitations and challenges, mainly because most conventional statistical tests do not account for the compositional nature of microbiota data, which may introduce biases when the compositional effects in a dataset are large. We therefore performed Mann-Whitney U (MWU) statistics on compositional taxonomic features, mainly to additionally statistically corroborate univariate signals as also identified by multivariate redundancyanalysis (RDA). This RDA approach (by the ‘Canoco’ method, and as also implemented in the ‘Vegan’ R-package, V2.6-8) performs a log-transformation on the compositional data prior to ordination, and is an established method in the field of microbiota research [24]. Lastly, some care should be taken when interpreting anastomotic healing scores (AHS) and histology, as human-based assessments carry a risk of biased results. However, the blinded assessment of histology by a certified pathologist, who evaluates basic pathology tasks such as determining ulceration, infiltration of eosinophils, granulation, and neutrophilic inflammation, is considered a sufficiently thorough method. Regarding the assessment of anastomotic healing, consistency and reliability are effectively addressed through evaluation by two different observers, with consensus required.

This is the first study to investigate the combined effect of common clinical preoperative and postoperative stressors on anastomotic healing and the gut microbiota in an experimental rat model. While we observe distinct changes in composition, there is growing evidence that individual function of microbes interferes with anastomotic healing. Further studies are needed to assess the impact of diclofenac in combination with WD on the function of the microbiome and virulence of individual species.

## 5. Conclusions

This study demonstrates that diclofenac in combination with an obesogenic Western diet causes gut microbiota alterations associated with macroscopic and microscopic evidence of disturbed anastomotic healing, of which findings are most prominent five days after surgery. 

## Data Availability

The datasets presented in this article are not readily available because they are part of an ongoing study. Requests to access the datasets should be directed to stijn.bluiminck1@radboudumc.nl.

## Supplementary Materials

The following supporting information can be downloaded at: https://www.mdpi.com/article/10.3390/biomedicines12102170/s1, Table S1. Comparative analysis of ingredients in Standard Diet (SD) and Western Diet (WD), Table S2. KEGG Pathway (KO-nrs) differential abundance analysis (DAA), Table S3. KEGG Orthology (K-nrs) differential abundance analysis (DAA), Figure S1. KEGG Pathway (KO-nrs) differential abundance analysis (DAA), Figure S2. KEGG Orthology (K-nrs) differential abundance analysis (DAA), Figure S3. KEGG PCA ordination analysis.
