# Supplementary material for: The Combined Effect of Western Diet Consumption and Diclofenac Administration Alters the Gut Microbiota and Promotes Anastomotic Leakage in the Distal Colon
Source: Biomedicines. 2024 Sep 24;12(10):2170. doi: 10.3390/biomedicines12102170 (PMC11504258; doi:10.3390/biomedicines12102170)
Supplement: Supplementary file 1 [file biomedicines-12-02170-s001.zip › biomedicines-3099025-supplementary.pdf]

Supplement 1

Table S1. Comparative analysis of ingredients in Standard Diet (SD) and Western Diet (WD)

##	Ingredients	SD	WD	##	Ingredients	SD	WD
**1. Fatty acids, gm/kg**	Total saturated	9	141	**10**	**Vitamins, mg/kg**		
	Total monounsaturated	13	162		Vitamin K3 (menadione)	50	0.52
	Total polyunsaturated	34	40.2		Vitamin B1 (thiamin)	17	3
					Vitamin B2 (riboflavin)	15	2.3
**2. Fat, %**		6.2	36		Niacin (nicotinic acid)	70	15
**3. Proteins, %**		18.6	20		Vitamin B6 (pyridoxine)	18	4.1
**4. Fiber, %**	Crude fiber	3.5	0		Pantothenic Acid	33	5.5
	Neutral detergent fiber	14.7	0		Vitamin B12 (cyanocobalamin)	0.08	0.04
					Folate	4	0.75
**5. Crude protein, %**		18.6	20.5		Choline	1200	1148
**6. Carbohydrates, %**	Simple sugars	3	22				
	Complex sugars	41	14	**11.**	**Vitamins, IU/kg**		
**7. Calories, kcal/gm**	Calories from protein	0.24	0.82		Vitamin A	15000	3162
	Calories from fat	0.18	3.24		Vitamin E	110	25.7
	Calories from carbohydrate	0.58	1.43				
**8. Minerals, gm/kg**	Calcium	10	5.6				
	Chloride	4	0.86				
	Phosphorus	7	5.8				
	Sodium	2	0.57				
	Potassium	6	5.6				
	Magnesium	2	0.49				
	Zinc	0.07	0.022				
	Manganese	0.1	0.047				
	Copper	0.015	0.004				
	Iodine	0.006	0.003				
	Iron	0.2	0.05				
**9. Amino Acids, gm/kg**	Aspartic Acid	14	12.8				
	Glutamic Acid	34	40.6				
	Alanine	11	5.3				
	Glycine	8	4.9				
	Threonine	7	8.7				
	Proline	16	20.5				
	Serine	11	11.4				
	Leucine	18	16.6				
	Isoleucine	8	11				
	Valine	9	13				
	Phenylalanine	10	8.9				
	Tyrosine	6	11.4				
	Methionine	4	7.1				
	Cystine	**3**	0.6				
	Lysine	9	14.8				
	Histidine	4	5.5				
	Arginine	10	7.3				
	Tryptophan	2	2.2				

Supplement 2

Methods:

16S rRNA Gene Sequencing and Data Preprocessing
A comparative analysis of gut microbiota functional profiles was conducted between two groups: 1) Western diet with diclofenac and 2) Western diet without diclofenac. The 16S rRNA gene sequencing data were processed using QIIME to generate an OTU table with absolute abundance data, which served as the input for functional prediction.

Functional Prediction with PICRUSt2
Functional predictions were performed using PICRUSt2 with default settings. PICRUSt2 utilized the 16S OTU table to infer the functional potential of microbial communities, generating data on KEGG Pathways (KO-nrs) and KEGG Orthology (K-nrs) features [51].

Differential Abundance Analysis (DAA) of KEGG Pathways
For the differential abundance analysis (DAA) of KEGG Pathway features, the Western diet with diclofenac group was compared to the Western diet without diclofenac group. The analysis was conducted using the R-package ggpicrust2 (v1.7.3). The default workflow function ggpicrust2(v1.7.3) was applied, employing the LinDA method for DAA and correcting for multiple testing using the Benjamini–Hochberg (BH) procedure. From a total of 212 KEGG Pathway features, only those with statistically significant differences were included in the final analysis. Visualization of these results was performed using the ggpicrust2() function's default plotting capabilities [52].

Differential Abundance Analysis (DAA) of KEGG Orthology (K-nrs)
A DAA on KEGG Orthology (K-nrs) features was also performed using the same R-package, ggpicrust2. For this analysis, the pathway_daa(), pathway_annotation(), and pathway_errorbar() functions were applied with default settings, except that the correction for multiple testing was adjusted to the Bonferroni method due to the high number of KEGG Orthology features. This stricter correction ensured a more conservative analysis, reducing the likelihood of false positives.

PCA Ordination Analysis of KEGG Pathways
To further explore the differences between the two groups, a PCA ordination analysis was conducted on the KEGG Pathway data. The pathway_pca() function from the ggpicrust2 package was used with default settings to generate PCA plots. Before conducting PCA, the KEGG Pathway data (i.e., the KO-nrs) required additional processing using the ko2kegg_abundance() function from the same R-package to appropriately aggregate the functional data.

.

Table S2 : KEGG Pathway (KO-nrs) differential abundance analysis (DAA): Contrast analysis (DAA) on KEGG Pathway features for HFD with/without diclofenac based on PICRUSt2 function predictions, listing only significant features (from n=212 total features).

#	feature	method	group1	group2	*p*_values	adj_ method	*p*_ adjust	pathway_name	pathway_class
**22**	ko04142	LinDA	HFD-C	HFD-D	2.85E-03	BH	0.046	*Lysosome*	*Cellular Processes; Transport and catabolism*
**23**	ko00604	LinDA	HFD-C	HFD-D	1.00E-04	BH	0.004	*Glycosphingolipid biosynthesis - ganglio series*	*Metabolism; Glycan biosynthesis and metabolism*
**28**	ko04973	LinDA	HFD-C	HFD-D	5.44E-04	BH	0.013	*Carbohydrate digestion and absorption*	*Organismal Systems; Digestive system*
**31**	ko04974	LinDA	HFD-C	HFD-D	3.86E-05	BH	0.004	*Protein digestion and absorption*	*Organismal Systems; Digestive system*
**57**	ko04962	LinDA	HFD-C	HFD-D	2.12E-03	BH	0.041	*Vasopressin-regulated water reabsorption*	*Organismal Systems; Excretory system*
**71**	ko00100	LinDA	HFD-C	HFD-D	6.14E-05	BH	0.004	*Steroid biosynthesis*	*Metabolism; Lipid metabolism*
**83**	ko00531	LinDA	HFD-C	HFD-D	2.01E-04	BH	0.005	*Glycosaminoglycan degradation*	*Metabolism; Glycan biosynthesis and metabolism*
**119**	ko00053	LinDA	HFD-C	HFD-D	1.34E-03	BH	0.028	*Ascorbate and aldarate metabolism*	*Metabolism; Carbohydrate metabolism*
**168**	ko00540	LinDA	HFD-C	HFD-D	1.70E-04	BH	0.005	*Lipopolysaccharide biosynthesis*	*Metabolism; Glycan biosynthesis and metabolism*
**176**	ko00140	LinDA	HFD-C	HFD-D	1.06E-04	BH	0.004	*Steroid hormone biosynthesis*	*Metabolism; Lipid metabolism*
**190**	ko00472	LinDA	HFD-C	HFD-D	2.74E-03	BH	0.046	*NA*	*NA*
**195**	ko04210	LinDA	HFD-C	HFD-D	1.69E-04	BH	0.005	*Apoptosis*	*Cellular Processes; Cell growth and death*
**198**	ko00944	LinDA	HFD-C	HFD-D	2.34E-05	BH	0.004	*Flavone and flavonol biosynthesis*	*Metabolism; Biosynthesis of other secondary metabolites*

Table S3 : KEGG Orthology (K-nrs) differential abundance analysis (DAA) :
Contrast analysis (DAA) on KEGG Orthology features for HFD with/without diclofenac based on PICRUSt2 function predictions, listing only significant features (from *n*=5591 total features).

#	feature	method	group1	group2	*p*_values	adj_method	*p*_adjust	description
**537**	K00895	LinDA	HFD-C	HFD-D	2.41E-07	bonferroni	0.001	*pfp, PFP; diphosphate-dependent phosphofructokinase [EC:2.7.1.90]*
**6408**	K18843	LinDA	HFD-C	HFD-D	3.88E-07	bonferroni	0.002	*hicB; antitoxin HicB*
**3862**	K07792	LinDA	HFD-C	HFD-D	4.76E-07	bonferroni	0.003	*dcuB; anaerobic C4-dicarboxylate transporter DcuB*
**2463**	K03841	LinDA	HFD-C	HFD-D	9.15E-07	bonferroni	0.006	*FBP, fbp; fructose-1,6-bisphosphatase I [EC:3.1.3.11]*
**4159**	K09181	LinDA	HFD-C	HFD-D	1.64E-06	bonferroni	0.011	*yfiQ; acetyltransferase*
**3398**	K07085	LinDA	HFD-C	HFD-D	1.85E-06	bonferroni	0.012	*K07085; putative transport protein*
**1268**	K02067	LinDA	HFD-C	HFD-D	2.75E-06	bonferroni	0.018	*mlaD, linM; phospholipid/cholesterol/gamma-HCH transport system substrate-binding protein*
**2735**	K05516	LinDA	HFD-C	HFD-D	2.81E-06	bonferroni	0.018	*cbpA; curved DNA-binding protein*
**1266**	K02065	LinDA	HFD-C	HFD-D	3.08E-06	bonferroni	0.020	*mlaF, linL, mkl; phospholipid/cholesterol/gamma-HCH transport system ATP-binding protein*
**1267**	K02066	LinDA	HFD-C	HFD-D	3.51E-06	bonferroni	0.023	*mlaE, linK; phospholipid/cholesterol/gamma-HCH transport system permease protein*
**117**	K00164	LinDA	HFD-C	HFD-D	3.56E-06	bonferroni	0.023	*OGDH, sucA; 2-oxoglutarate dehydrogenase E1 component [EC:1.2.4.2]*
**543**	K00912	LinDA	HFD-C	HFD-D	3.61E-06	bonferroni	0.024	*lpxK; tetraacyldisaccharide 4′-kinase [EC:2.7.1.130]*
**2221**	K03578	LinDA	HFD-C	HFD-D	3.66E-06	bonferroni	0.024	*hrpA; ATP-dependent helicase HrpA [EC:3.6.4.13]*
**6217**	K18138	LinDA	HFD-C	HFD-D	3.69E-06	bonferroni	0.024	*acrB, mexB, adeJ, smeE, mtrD, cmeB; multidrug efflux pump*
**4102**	K09007	LinDA	HFD-C	HFD-D	3.74E-06	bonferroni	0.025	*folE2; GTP cyclohydrolase IB [EC:3.5.4.16]*
**5306**	K13628	LinDA	HFD-C	HFD-D	3.95E-06	bonferroni	0.026	*iscA; iron-sulfur cluster assembly protein*
**6218**	K18139	LinDA	HFD-C	HFD-D	4.00E-06	bonferroni	0.026	*oprM, emhC, ttgC, cusC, adeK, smeF, mtrE, cmeC, gesC; outer membrane protein, multidrug efflux system*
**23**	K00029	LinDA	HFD-C	HFD-D	4.08E-06	bonferroni	0.027	*E1.1.1.40, maeB; malate dehydrogenase (oxaloacetate-decarboxylating)(NADP+) [EC:1.1.1.40]*
**3861**	K07791	LinDA	HFD-C	HFD-D	4.13E-06	bonferroni	0.027	*dcuA; anaerobic C4-dicarboxylate transporter DcuA*
**2785**	K05595	LinDA	HFD-C	HFD-D	4.25E-06	bonferroni	0.028	*marC; multiple antibiotic resistance protein*
**757**	K01261	LinDA	HFD-C	HFD-D	2.65E-06	bonferroni	0.017	*pepA; glutamyl aminopeptidase [EC:3.4.11.7]*

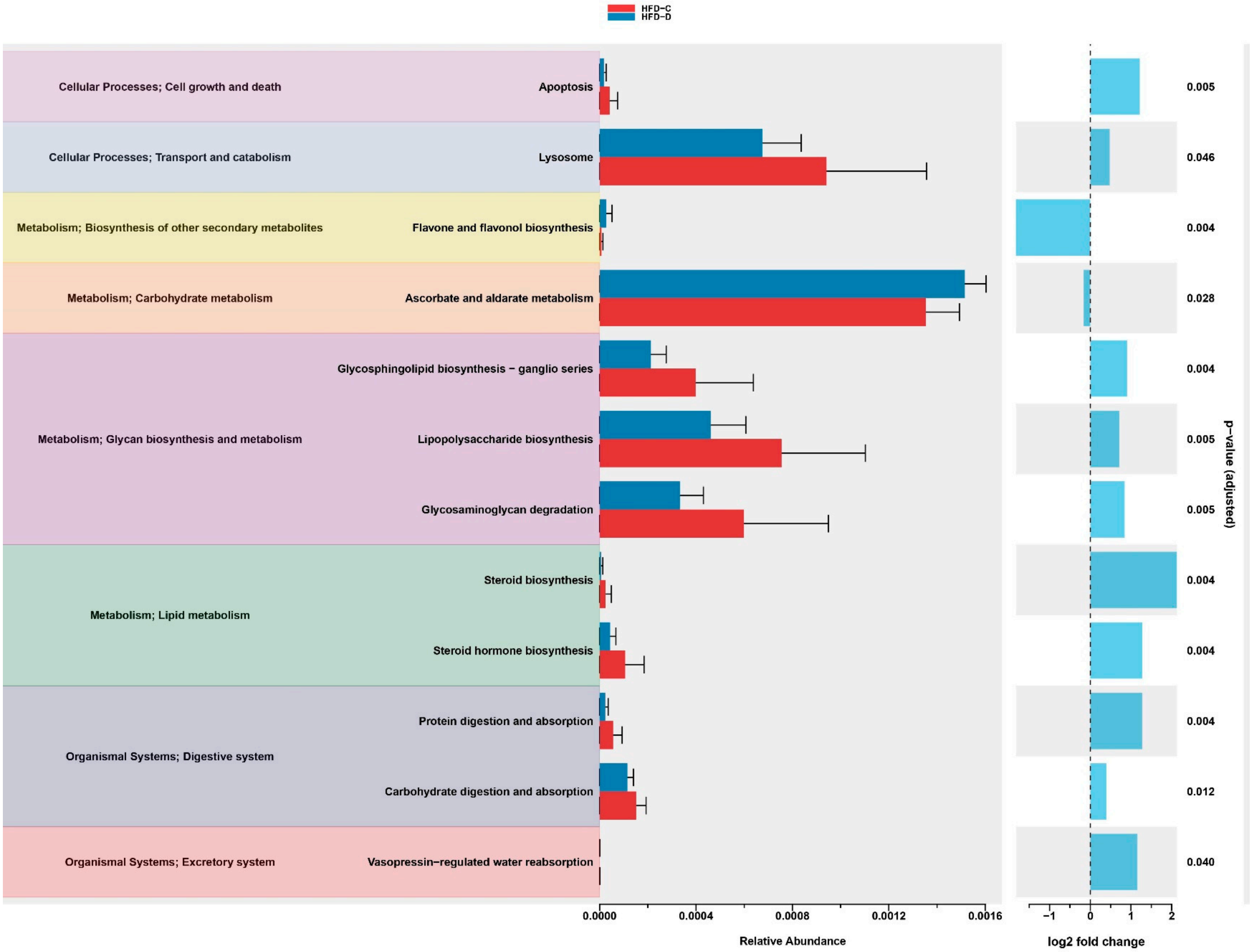

Figure S1. KEGG Pathway (KO-nrs) differential abundance analysis (DAA) :

Contrast analysis (DAA) on KEGG Pathway features for HFD with/without diclofenac based on PICRUSt2 function predictions. Figure shows only the significant features (from *n*=212 total features). In red are HFD control samples (HFD-C) and in blue are HFD samples with diclofenac (HFD-D). For more details on these features, see Table S1. The data were generated by PICRUSt2 (default settings) on the 16S OTU-table with absolute abundance data as derived from QIIME. Further analysis and visualization of the PICRUSt2 output data was performed with the R-package '*ggpicrust2*' (v1.7.3) by applying its default workflow function called *ggpicrust2()* and selecting default settings (i.e., DAA method by LinDA, and correction for multiple testing by BH (Benjamini–Hochberg)).

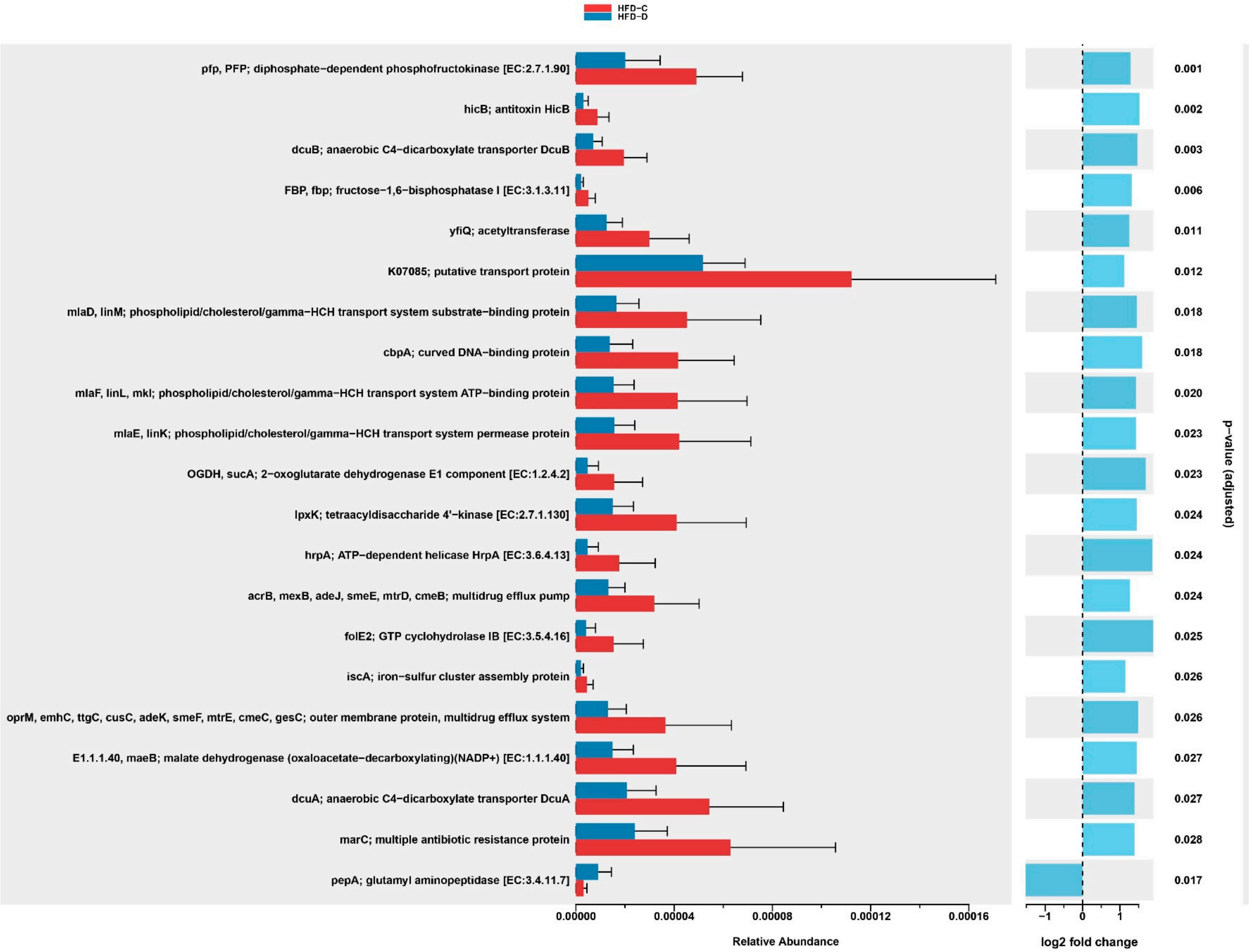

Figure S2. KEGG Orthology (K-nrs) differential abundance analysis (DAA) :

Contrast analysis (DAA) on KEGG Orthology features for HFD with/without diclofenac based on PICRUSt2 function predictions. Figure shows only the significant features (from *n*=5591 total features). In red are HFD control samples (HFD-C) and in blue are HFD samples with Diclofenac (HFD-D). For more details on these features, see Table S2.

The data were generated by PICRUSt2 (default settings) on the 16S OTU-table with absolute abundance data as derived from QIIME. Further analysis and visualization of the PICRUSt2 output data was performed with the R-package '*ggpicrust2*' (v1.7.3) by applying its functions called *pathway_daa()*, *pathway_annotation()*, and *pathway_errorbar()* and selecting default settings (i.e., DAA method by LinDA), except for here selecting a more stringent correction for multiple testing by Bonferroni because of the very high number of KEGG Orthology (K-nrs) features.

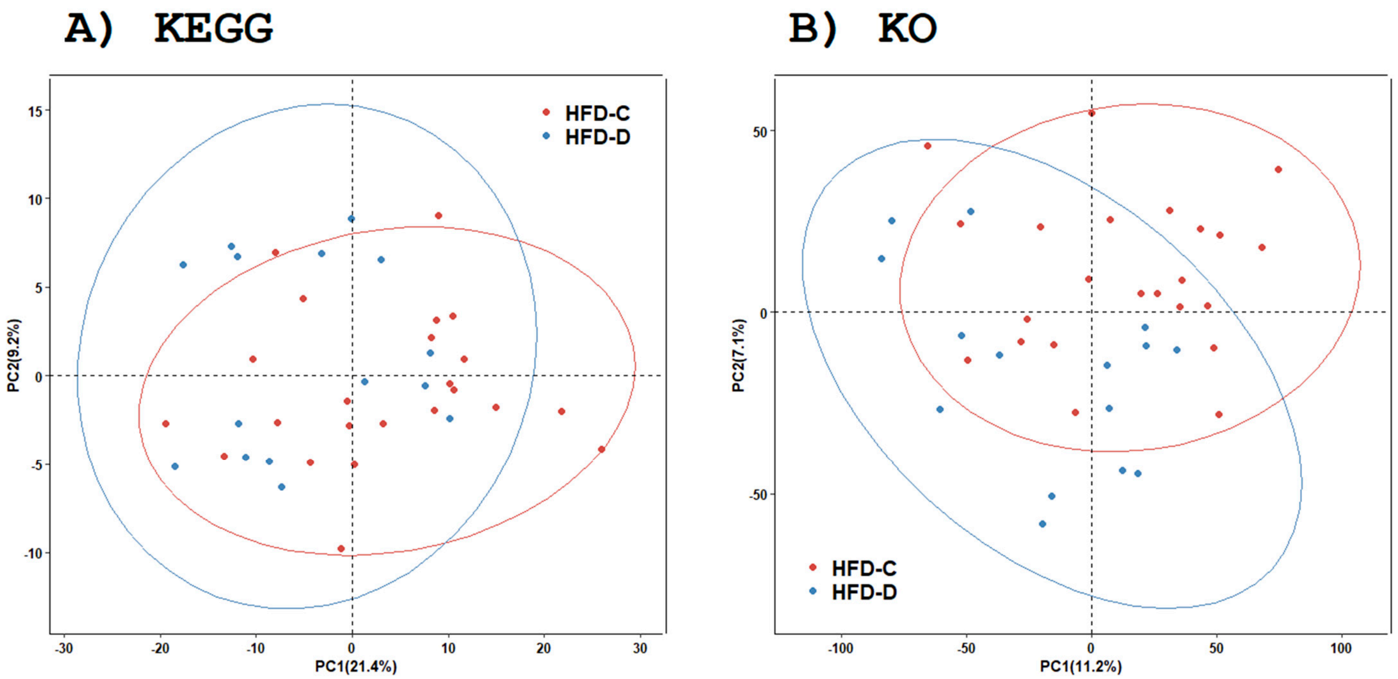

Figure S3 : KEGG PCA ordination analysis : PCA was performed on (**A**) KEGG Pathway (KO-nrs) features and (**B**) KEGG Orthology (K-nrs) features for HFD (i.e., Western diet) with/without diclofenac based on PICRUSt2 function predictions. In red are HFD control samples (HFD-C) and in blue are HFD samples with Diclofenac (HFD-D). For creation of these PCA figures, the R-package '*ggpicrust2*' was used (v1.7.3) by applying its function called *pathway_pca()* with default settings. Note that generating the KEGG Pathway data (i.e., the KO-nrs) required an additional processing step with the *ko2kegg_abundance()* function as included in the same R-package.
